# Synthesis, Crystal Structure, and Biological Activity of Ethyl 4-Methyl-2,2-dioxo-1*H*-2λ^6^,1-benzothiazine-3-carboxylate Polymorphic Forms

**DOI:** 10.3390/scipharm86020021

**Published:** 2018-05-30

**Authors:** Igor V. Ukrainets, Anna A. Burian, Vyacheslav N. Baumer, Svitlana V. Shishkina, Lyudmila V. Sidorenko, Igor A. Tugaibei, Natali I. Voloshchuk, Pavlo S. Bondarenko

**Affiliations:** 1 Department of Pharmaceutical Chemistry, National University of Pharmacy, 53 Pushkinska st., 61002 Kharkiv, Ukraine; anna_chem@ukr.net (A.A.B.); slv.ludmila@i.ua (L.V.S.); 2 SSI “Institute for Single Crystals”, National Academy of Sciences of Ukraine, 60 Nauki ave., 61001 Kharkiv, Ukraine; baumer@xray.isc.kharkov.com (V.N.B.); sveta@xray.isc.kharkov.com (S.V.S.); 3 Department of Inorganic Chemistry, V. N. Karazin Kharkiv National University, 4 Svobody sq., 61077 Kharkiv, Ukraine; 4 Department of Clinical Biochemistry, Forensic Toxicology, and Pharmacy, Kharkiv Medical Academy of Postgraduate Education, 58 Amosov st., 61176 Kharkiv, Ukraine; igor_doctor@rambler.ru; 5 Department of Pharmacology, N. I. Pirogov Vinnitsa National Medical University, 56 Pirogov st., 21018 Vinnitsa, Ukraine; voloshchuknatali@gmail.com (N.I.V.); bondarenkopavel1506@gmail.com (P.S.B.)

**Keywords:** alkylation, esters, 4-methyl-2,2-dioxo-1*H*-2λ^6^,1-benzothiazine-3-carboxylic acid, 2,1-benzothiazine, crystal structure, polymorphism, anti-inflammatory action, analgesic activity

## Abstract

Continuing the search for new potential analgesics among the derivatives of 4-methyl-2,2-dioxo-1*H*-2λ^6^,1-benzothiazine-3-carboxylic acid, the possibility of obtaining its esters by the alkylation of the corresponding sodium salt with iodoethane in dimethyl sulfoxide (DMSO) at room temperature was studied. It was found that under such conditions, together with the oxygen atom of the carboxyl group, a heteroatom of nitrogen is also alkylated. Therefore, the product of the reaction studied is a mixture of ethyl 4-methyl-2,2-dioxo-1*H*-2λ^6^,1-benzothiazine-3-carboxylate (major) and its 1-ethyl-substituted analog (minor). A simple but very effective method of preparative separation of these compounds was proposed. Moreover, the heterogeneous crystallization from ethanol was revealed to result in a monoclinic polymorphic form of ethyl 4-methyl-2,2-dioxo-1*H*-2λ^6^,1-benzothiazine-3-carboxylate, while the homogeneous crystallization results in its orthorhombic form. The molecular and crystal structures of both forms were confirmed by X-ray diffraction analysis, and the phase purity by powder diffraction study. The pharmacological tests carried out on the model of a carrageenan edema showed that the screening dose of 20 mg/kg of 1-ethyl-substituted ester and the orthorhombic form of its analog unsubstituted in position 1 exhibited weak anti-inflammatory and moderate analgesic effects. At the same time, the monoclinic form of ethyl 4-methyl-2,2-dioxo-1*H*-2λ^6^,1-benzothiazine-3-carboxylate appeared to be both a powerful analgesic and an anti-inflammatory agent that exceeded Piroxicam and Meloxicam in the same doses by these indicators. A detailed comparative analysis of the molecular and crystal structures of two polymorphic forms of ethyl 4-methyl-2,2-dioxo-1*H*-2λ^6^,1-benzothiazine-3-carboxylate was carried out using quantum chemical calculations of the energies of pairwise interactions between molecules. An explanation of the essential differences of their biological properties based on this was offered.

## 1. Introduction

Ester compounds as officially permitted drugs are widely represented in practically all pharmacological groups. Agents for the control of pain and pain syndromes of different etiologies are no exception. More than 50 synthetic analgesics with a central and peripheral action, non-steroidal anti-inflammatory drugs, as well as local anesthetics, belong to ester compounds due to their chemical structure [1,2].

Ester compounds attract the attention of scientists engaged in a targeted search for new biologically active substances because of their high efficiency and relatively small number of side effects [3]. They are also very convenient for this kind of research because of the fact that any of their components (both acid and alcoholic) can be easily modified if necessary. Moreover, these compounds are almost unlimited and, last but not least, they are well-studied using repeatedly tested methods. As another positive point, it should be noted that esterases that are responsible for the biodegradation of such drugs are present in all human organs and tissues in large quantities [4]. Therefore, the risk of an “overload” of the enzyme systems of a patient with a drug ester is reduced to a minimum [3].

**Figure 1:**
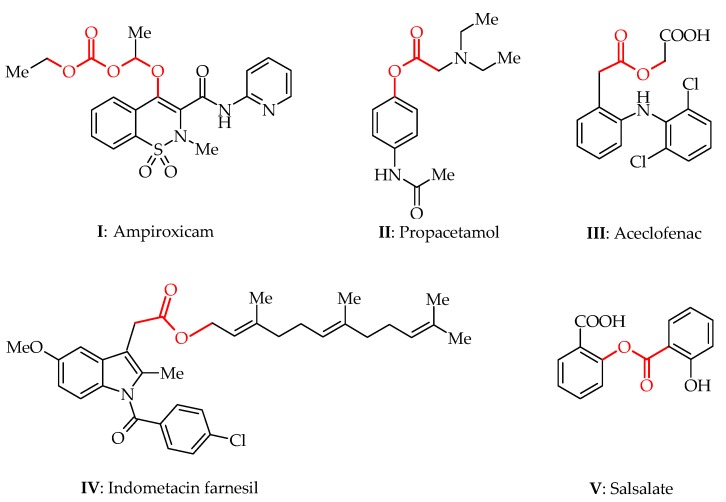
The ester-prodrugs of analgesic acids and phenols that are officially registered drugs [1,2].

Often, the transformation of a group with acid properties in the ester group leads to changes in the pharmacokinetic, pharmaceutical, and/or pharmacological properties of drug acids in a desired direction. Therefore, it is not surprising that this property is widely used to transform pharmaceutical acids, alcohols, or phenols of different pharmacological orientations (including analgesics, which are well-proven in medical practice (Figure 1)) into more suitable and safer ester prodrugs [1,2,3]. At the same time, enol (**I**) or phenolic (**II**) hydroxyl, and the carboxyl group (**III** and **IV**), as well as phenolic hydroxyl and the COOH-group (**V**), can be successfully subjected to modification.

**Scheme 1:**
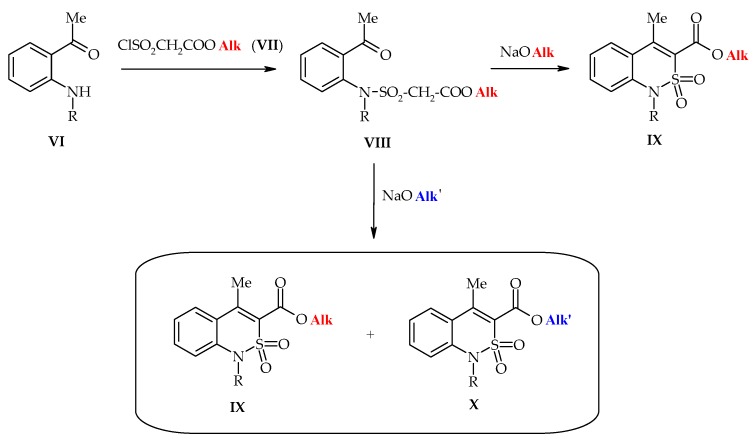
The conventional method for the synthesis of alkyl 1-R-4-methyl-2,2-dioxo-1*H*-2λ^6^,1-benzothiazine-3-carboxylates **IX [22,23,24,25]**.

To date, organic chemistry has an extremely wide range of methods and reagents that provides for the gain of ester compounds virtually without any restrictions [5,6,7,8,9,10,11,12,13,14,15,16,17,18,19,20,21]. In principle, modern pharmaceutical chemistry is capable of applying all the advanced scientific achievements in this field. However, in real industrial-scale syntheses, as a rule, manufacturers are guided not only by the novelty of technologies, but also by their safety (primarily environmental), reliability, and economic feasibility. Therefore, in the schemes for obtaining drug esters, the time-tested methods for the formation of ester fragments are the most common. These are Fisher esterification—a generally recognized method—and the acylation of alcohols (phenols) with anhydrides or halides of carboxylic acids [1]. In some cases, another traditional version of the synthesis of ester compounds (the alkylation of carboxylic acid salts with alkyl halides) worked well. This is the method that we used to transform 4-methyl-2,2-dioxo-1*H*-2λ^6^,1-benzothiazine-3-carboxylic acid into an ester. Naturally, the lower alkyl esters of this acid are easier to obtain according to the usual linear scheme [22,23] when the required substituent is introduced at the preliminary stage of the synthesis of the corresponding alkyl (chlorosulfonyl) acetate **VII** (Scheme 1). It must be remembered that the use of bases at the final stage of the hetericyclization provides an opportunity to avoid easy transesterification [24,25].

However, we deliberately focused on the alkylation of the benzothiazine-3-carboxylic acid salt with the alkyl halide in order to involve the most diverse esters in the alcoholic part of the molecule, in addition to the already studied methyl 4-methyl-2,2-dioxo-1*H*-2λ^6^,1-benzothiazine-3-carboxylates [23]. It is the alkylation of the salts of the carboxylic acids with the alkyl halides characterized by a very wide synthetic potential (unlike the method presented in Scheme 1) that allows for the solution of similar problems. In addition, the reactions of the alkylation of ambident nucleophiles are always interesting from a theoretical point of view since, in most cases, they are ambiguous and not always predictable.

## 2. Materials and Methods

### 2.1. Chemistry

^1^H-NMR (proton nuclear magnetic resonance) spectra were acquired on a Varian Mercury-400 (Varian Inc., Palo Alto, CA, USA) instrument (400 MHz) in hexadeutero-dimethyl sulfoxide (DMSO-d_6_) with tetramethylsilane as the internal standard. The chemical shift values were recorded on a *δ* scale and the coupling constants (*J*) in hertz. The following abbreviations were used in reporting the spectra: s = singlet, d = doublet, t = triplet, q = quartet. The melting points were determined in a capillary using a electrothermal IA9100X1 (Bibby Scientific Limited, Stone, UK) digital melting point apparatus. The synthesis of the starting sodium 4-methyl-2,2-dioxo-1*H*-2λ^6^,1-benzothiazine-3-carboxylate monohydrate (**1**) was carried out by the method described in [26].

### 2.2. Monoclinic Form of Ethyl 4-Methyl-2,2-dioxo-1H-2λ^6^,1-benzothiazine-3-carboxylate (2M)

Iodoethane (1.56 g, 0.01 mol) was added to the solution of sodium salt **1** (2.79 g, 0.01 mol) in 10 mL dimethyl sulfoxide (DMSO), and the mixture was stirred for 5 h at 25°C. The mixture was diluted with cold water and acidified with dilute HCl to рH 3. The precipitate formed was filtered, washed with cold water, and dried in the air. A total of 2.00 g of the product composed of ester **2** and its *N*-ethyl-substituted analog **3** (according to the data of the ^1^H-NMR spectrum—68 and 32%, respectively) was isolated. The mixture of the crystals of esters **2M** and **3** obtained by the crystallization from ethanol in 10 mL of hexane was boiled for 5 min, then the hexane was carefully decanted, avoiding the sediment. The procedure was repeated five times. Finally, a pure monoclinic form of **2M** was obtained. The yield was: 1.26 g (47%); colorless crystals; melting point (mp) 171–173 °C; ^1^H-NMR (400 MHz, DMSO-*d*_6_): δ 11.83 (br. s, 1H, SO_2_NH), 7.78 (d, 1H, *J* = 8.0 Hz, H-5), 7.50 (t, 1H, *J* = 7.7 Hz, H-7), 7.22 (t, 1H, *J* = 7.7 Hz, H-6), 7.12 (d, 1H, *J* = 8.0 Hz, H-8), 4.31 (q, 2H, *J* = 7.1 Hz, OCH_2_), 2.43 (s, 3H, 4-CH_3_), 1.27 (t, 3H, *J* = 7.1 Hz, OCH_2_CH_3_). This was analytically calculated (Anal.Calcd.) for C_12_H_13_NO_4_S: C, 53.92; H, 4.90; N, 5.24; S, 12.00. We found: C, 53.98; H, 4.99; N, 5.17; S, 11.91.

The filtrate obtained after the isolation of esters **2** and **3** was kept for several days at room temperature. Gradually, crystals of the product identified by the ^1^H-NMR spectrum and the absence of the depression of the melting point of the sample mixed with the known reference 4-methyl-2,2-dioxo-1*H*-2λ^6^,1-benzothiazine-3-carboxylic acid monohydrate [26] formed in the solution.

### 2.3. Orthorhombic Form of Ethyl 4-Methyl-2,2-dioxo-1H-2λ^6^,1-benzothiazine-3-carboxylate (2O)

The mixture of esters **2** and **3** obtained was treated after the alkylation of salt **1** (see the previous example) with 15 mL of 10% aqueous solution of Na_2_CO_3_, and filtered. The filtrate was acidified with dilute HCl to рH 3. The precipitate of the pure ester **2** formed was filtered, washed with cold water, and dried in the air. After the crystallization from ethanol, an orthorhombic form **2O** was obtained. The yield was: 1.31 g (49%); colorless crystals; m.p. 165–167 °C; ^1^H-NMR (400 MHz, DMSO-*d*_6_): δ 7.84 (d, 1H, *J* = 8.0 Hz, H-5), 7.61 (t, 1H, *J* = 7.8 Hz, H-7), 7.51 (d, 1H, *J* = 7.9 Hz, H-8), 7.33 (t, 1H, *J* = 7.8 Hz, H-6), 4.32 (q, 2H, *J* = 7.0 Hz, OCH_2_), 3.96 (q, 2H, *J* = 7.1 Hz, NCH_2_), 2.43 (s, 3H, 4-CH_3_), 1.27 (t, 3H, *J* = 7.0 Hz, OCH_2_CH_3_), 1.14 (t, 3H, *J* = 7.1 Hz, NCH_2_CH_3_). The Anal.Calcd. was C_12_H_13_NO_4_S: C, 53.92; H, 4.90; N, 5.24; S, 12.00. We found: C, 54.00; H, 4.96; N, 5.31; S 11.93%.

### 2.4. Ethyl 1-Ethyl-4-methyl-2,2-dioxo-1H-2λ^6^,1-benzothiazine-3-carboxylate (3)

The residue was washed on the filter (see the previous example) undissolved in an aqueous solution of Na_2_CO_3_ with cold water, and dried in the air. The yield was: 0.67 g (23%); colorless crystals; m.p. 96–98 °C (ethanol); ^1^H-NMR (400 MHz, DMSO-*d*_6_): δ 7.84 (d, 1H, *J* = 8.0 Hz, H-5), 7.61 (t, 1H, *J* = 7.8 Hz, H-7), 7.51 (d, 1H, *J* = 7.9 Hz, H-8), 7.33 (t, 1H, *J* = 7.8 Hz, H-6), 4.32 (q, 2H, *J* = 7.0 Hz, OCH_2_), 3.96 (q, 2H, *J* = 7.1 Hz, NCH_2_), 2.43 (s, 3H, 4-CH_3_), 1.27 (t, 3H, *J* = 7.0 Hz, OCH_2_CH_3_), 1.14 (t, 3H, *J* = 7.1 Hz, NCH_2_CH_3_). The Anal.Calcd. was C_14_H_17_NO_4_S: C, 56.93; H, 5.80; N, 4.74; S 10.86%. We found: C, 56.85; H, 5.74; N, 4.82; S 10.95%.

### 2.5. X-ray Structural Analysis of Ethyl 4-Methyl-2,2-dioxo-1H-2λ^6^,1-benzothiazine-3-carboxylate Monoclinic Form (2M)

The crystals of ester **2M** (C_12_H_13_NO_4_S) were monoclinic and colorless at 293 K: *a* 8.0177(6), *b* 10.259(1), *c* 7.4995(9) Å; β 90.130(2)°; *V* 616.9(1) Å^3^, *Z* 2, space group Pc, *d*_calc_ 1.439 g/cm^3^, µ(MoK_α_) 0.268 mm^−1^, *F*(000) 294. The unit cell parameters and intensities of 6074 reflections (3187 independent reflections, *R*_int_ = 0.073) were measured on an Xcalibur-3 diffractometer (Oxford Diffraction Limited, Oxford, UK) using MoK_α_ radiation, a charge-coupled device (CCD) detector, graphite monochromator, and ω-scanning to 2θ_max_ 60°. The structure was solved by the direct method using the SHELXTL program package (Institute of Inorganic Chemistry, Göttingen, Germany) [27]. The positions of the hydrogen atoms were found from the electron density difference map and refined using the riding model with *U*_iso_ = *nU*_eq_ for the non-hydrogen atom bonded to a given hydrogen atom (*n* = 1.5 for methyl groups, and *n* = 1.2 for the other hydrogen atoms). The hydrogen atom at the N_(1)_ atom was refined using isotropic approximation. The structure was refined using *F*^2^ full-matrix least-squares analysis in the anisotropic approximation for non-hydrogen atoms to *wR*_2_ 0.136 for 3,118 reflections (*R*_1_ 0.054 for 2,350 reflections with *F* > 4σ(*F*), *S* 0.989). The final atomic coordinates, and the crystallographic data for the molecule of ester **2M** have been deposited to with the Cambridge Crystallographic Data Centre, 12 Union Road, CB2 1EZ, UK (fax: +44-1223-336033; e-mail: deposit@ccdc.cam.ac.uk) and are available on request quoting the deposition number CCDC 1835802 [28].

### 2.6. X-ray Structural Analysis of Ethyl 4-Methyl-2,2-dioxo-1H-2λ^6^,1-benzothiazine-3-carboxylate Orthorhombic Form (2O)

The crystals of ester **2O** (C_12_H_13_NO_4_S) were orthorhombic and colorless at 293 K: *a* 8.957(1), *b* 15.866(3), *c* 17.955(3) Å; *V* 2551.7(8) Å^3^, *Z* 8, space group Pbca, *d*_calc_ 1.392 g/cm^3^, µ(MoK_α_) 0.260 mm^−1^, *F*(000) 1120. The unit cell parameters and intensities of 23,700 reflections (3717 independent reflections, *R*_int_ = 0.159) were measured on an Xcalibur-3 diffractometer (Oxford Diffraction Limited) using MoK_α_ radiation, a CCD detector, graphite monochromator, and ω-scanning to 2θ_max_ 60°. The structure was solved by the direct method using the SHELXTL program package (Institute of Inorganic Chemistry) [27]. The positions of the hydrogen atoms were found from the electron density difference map and refined using the riding model with *U*_iso_ = *nU*_eq_ for the non-hydrogen atom bonded to a given hydrogen atom (*n* = 1.5 for methyl groups, and *n* = 1.2 for the other hydrogen atoms). The hydrogen atom at the N_(1)_ atom was refined using isotropic approximation. The structure was refined using *F*^2^ full-matrix least-squares analysis in the anisotropic approximation for non-hydrogen atoms to 0.190 for 3,675 reflections (*R*_1_ 0.070 for 1880 reflections with *F* > 4σ(*F*), *S* 0.889). The final atomic coordinates, and the crystallographic data for the molecule of ester **2R** have been deposited to with the Cambridge Crystallographic Data Centre, 12 Union Road, CB2 1EZ, UK (fax: +44-1223-336033; e-mail: deposit@ccdc.cam.ac.uk) and are available on request quoting the deposition number CCDC 1835803 [29].

### 2.7. X-ray Structural Analysis of Ethyl 1-Ethyl-4-methyl-2,2-dioxo-1H-2λ^6^,1-benzothiazine-3-carboxylate (3)

The crystals of the *N*-ethyl substituted ester **3** (C_14_H_17_NO_4_S) were triclinic and colorless at 293 K: *a* 10.2546(7), *b* 11.3609(9), *c* 13.1329(9) Å; β 105.068(6)°; γ 100.765(6)°; *V* 1433.3(2) Å^3^, *Z* 4, space group P$\bar{1}$, *d*_calc_ 1.369 g/cm^3^, µ(MoK_α_) 0.238 mm^−1^, *F*(000) 624. The unit cell parameters and intensities of 14,670 reflections (8344 independent reflections, *R*_int_ = 0.030) were measured on an Xcalibur-3 diffractometer (Oxford Diffraction Limited) using MoK_α_ radiation, a CCD detector, graphite monochromator, and ω-scanning to 2θ_max_ 60°. The structure was solved by the direct method using the SHELXTL program package (Institute of Inorganic Chemistry) [27]. The positions of the hydrogen atoms were found from the electron density difference map and refined using the “rider” model with *U*_iso_ = *nU*_eq_ for the nonhydrogen atom bonded to a given hydrogen atom (*n* = 1.5 for methyl groups, and *n* = 1.2 for the other hydrogen atoms). The structure was refined using *F*^2^ full-matrix least-squares analysis in the anisotropic approximation for non-hydrogen atoms to *wR*_2_ 0.145 for 8,264 reflections (*R*_1_ 0.051 for 5404 reflections with *F* > 4σ(*F*), *S* 0.967). The final atomic coordinates, and the crystallographic data for the molecule of ester **3** have been deposited to with the Cambridge Crystallographic Data Centre, 12 Union Road, CB2 1EZ, UK (fax: +44-1223-336033; e-mail: deposit@ccdc.cam.ac.uk) and are available on request quoting the deposition number CCDC 1835804 [30].

### 2.8. Powder Diffraction Study of Ethyl 4-Methyl-2,2-dioxo-1H-2λ^6^,1-benzothiazine-3-carboxylate Monoclinic and Orthorhombic Form (2M and 2O)

The powder diffraction study of the monoclinic and orthorhombic forms of ethyl 4-methyl-2,2-dioxo-1*H*-2λ^6^,1-benzothiazine-3-carboxylate (**2M** and **2O**) was performed using a Siemens D500 diffractometer (Siemens Analytical X-ray Instruments Inc., Madison, WI, USA) with Bragg–Brentano geometry, a curved graphite monochromator on the counter arm, scanned with Δ2θ = 0.02°, with an accumulation time at each point of 30 s. The Rietveld refinement was carried out with the WinPLOTR and FullProf programs (Institute Laue-Langevin, Grenoble, France) [31,32,33] using the model structures obtained by the monocrystal diffraction method.

### 2.9. Pharmacology

#### Anti-Inflammatory and Analgesic Tests

All biological experiments were carried out in full accord with the European Convention on the Protection of Vertebrate Animals Used for Experimental and Other Scientific Purposes and Ukrainian Law No. 3447-IV “On the protection of animals from severe treatment” [34] (project ID 3410U14, approved 15 October 2015).

In this study, male Wistar rats (200–250 g) were obtained from vivarium of the Institute of Pharmacology and Toxicology of the Academy of Medical Sciences of Ukraine (Kiev, Ukraine). All animals received standard food for rodents and water. They were acclimatized within 10 days. One day before the experiments, the animals were transferred to the scientific laboratory for adaptation. All the time they were maintained at 20–22 °C, 40–60% relative humidity and a 12 h/12 h (light/dark) cycle.

The anti-inflammatory action of the ester **2** two polymorphic modifications and ester **3** was studied on the model of the experimental inflammation process caused by subplantar introduction of 0.1 mL of 1% carrageenan solution [35,36] in one of the hind limbs of the rats. The test compounds and the reference drugs were injected intraperitoneally into the animals of the experimental groups 2 h after the introduction of carrageenan. Three hours after the carrageenan injection (at the peak of inflammation), the volume of healthy and swollen limbs (mm^3^) was measured using a plethysmometer (Ugo Basile Biological Research Apparatus Company, Varese, Italy). The anti-inflammatory effect (in %) was assessed by the degree of edema reduction in the experimental animals compared to the rats in the control group.

The analgesic action was detected on the same model by determining the pain threshold—the smallest pressure force (g/mm^2^) on the rat’s foot that caused a painful reaction of the animal, expressed by localization of pain and/or paw withdrawal—the healthy and the injured limb. The measurements were performed using a baseline dolorimeter (Fabrication Enterprises, White Plains, NY, USA). The analgesic effect (in %) of the compounds studied was assessed by the ability to increase the pain threshold in the experimental groups compared to the rats in the control group.

All test substances, Piroxicam (Jenapharm, Jena, Germany) and Meloxicam (Boehringer Ingelheim, Ingelheim am Rhein, Germany), were introduced intraperitoneally in the form of fine aqueous suspensions stabilized with Tween-80 at a screening dose of 20 mg/kg. The animals of the control group received an equivalent amount of water with Tween-80.

Seven experimental animals were involved to obtain statistically reliable results in testing each of the esters **2M**, **2O** and **3**, the reference drugs and the control. The processing of the results obtained was performed using the STATISTICA 6.1 software package (StatSoft Inc., Tulsa, OK, USA). Descriptive statistics included calculations of the arithmetic means (M) and the standard errors of the mean (±m). The significance of the differences within one group was found using Wilcoxon’s non-parametric test. The reliability of intergroup differences was determined using non-parametric Mann–Whitney *U*-criteria. Effects were regarded as statistically significant at *p* ≤ 0.05.

### 2.10. Quantum-Chemical Calculations

The packing analysis of the **2M** and **2O** polymorphic forms was performed within an energetic approach that has been suggested earlier [37]. The first coordination sphere of the crystal building unit (molecule or dimer of molecules) was determined using the “Molecular Shell calculation” option within the Mercury program, version 3.8 (Cambridge Crystallographic Data Centre, Cambridge, UK) [38]. The molecular geometries of the building unit dimers were taken from X-ray diffraction studies. Taking into account the well-known effect of shortening the lengths of the X-H bonds in the X-ray diffraction data [39], the positions of the hydrogen atoms were normalized according to the results of the geometry optimization of the isolated molecule. Other bonds and torsion angles were not changed.

The pairwise interaction energies were calculated using the B97-D3/Def2-TZVP density functional method [40,41,42] and corrected for the basis set superposition error with the counterpoise method [43]. All calculations were performed using ORCA 3.0 software (Max Planck Institute, Mülheim an der Ruhr, Germany) [44]. The results of the calculations were visualized with energy-vector diagrams [37], where the vector lengths were calculated using the following equation: L_i_ = (R_i_E_i_)/2E_str_, where R_i_ is a distance between the geometrical centers of the interacting molecules, E_i_ is the energy of interaction between these two molecules and E_str_ is the energy of the strongest pairwise interaction in the crystal. Such a diagram is a molecule image and may be multiplied with all symmetry operations similar to a molecule.

## 3. Results and Discussion

### 3.1. Chemistry

**Scheme 2:**
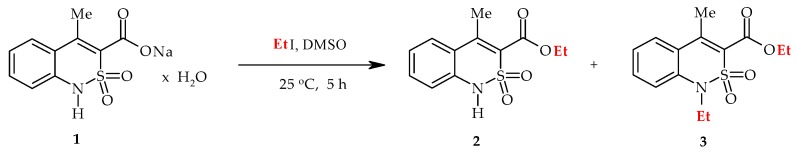
Alkylation of sodium 4-methyl-2,2-dioxo-1*H*-2λ^6^,1-benzothiazine-3-carboxylate monohydrate (1) (Et = C_2_H_5_).

The reaction of the sodium 4-methyl-2,2-dioxo-1*H*-2λ^6^,1-benzothiazine-3-carboxylate monohydrate (**1**) and the equivalent amount of iodoethane in the DMSO solution proceeds rather easily at room temperature. The ^1^H-NMR spectrum of the crude product showed that, as expected, alkylation did not clearly proceed. Along with the expected ethyl ester **2**, its *N*-ethyl-substituted analog **3** was also formed, and in a fairly large amount—approximately 36% (Scheme 2). It is of interest that the original product was naturally present (not in the form of salt **1**, but in the corresponding 4-methyl-2,2-dioxo-1*H*-2λ^6^,1-benzothiazine-3-carboxylic acid) in the reaction mixture. However, we failed to detect the product of monoalkylation solely by the nitrogen heteroatom. Hence, the ethyl iodide initially attacks the oxygen atom of the carboxylate anion. However, while ethyl ester **2** was forming as a new potential target for electrophilic attack, it began to seriously compete with salt **1**. As a result, there was a relatively high content of *O,N*-dialkylation product **3**.

**Figure 2:**
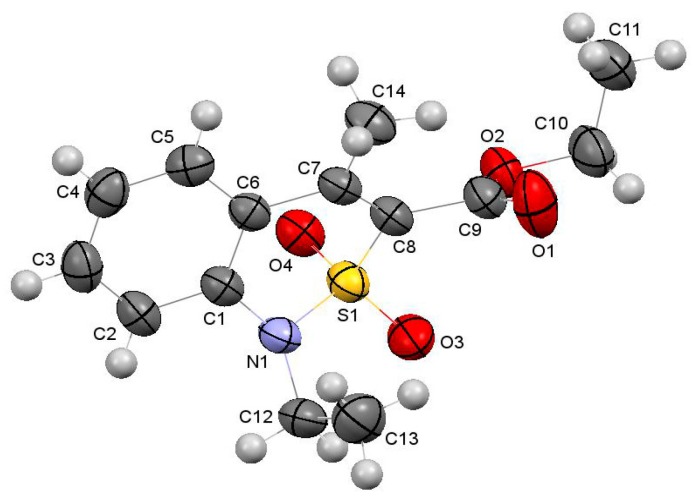
The spatial structure of 1-ethyl substituted ester **3** according to the X-ray diffraction data. The atoms are represented by thermal vibration ellipsoids of 50% probability.

With regard to their solubility in ethanol, esters **2** and **3** differ insignificantly, therefore, it is not possible to separate them by conventional crystallization from this solvent. However, quite unexpectedly, much more interesting results were obtained than the trivial separation of the reaction mixture into its individual components. Thus, the crystallization of a crude product from ethanol produced two outwardly different types of crystals: parallelepipeds and thick plates. According to the X-ray structural analysis, the first of them—parallelepipeds—appeared to be ethyl 1-ethyl-4-methyl-2,2-dioxo-1*H*-2λ^6^,1-benzothiazine-3-carboxylate (**3**) (Figure 2).

There were two molecules (**A** and **B**) of this compound that differed in some geometrical parameters in the asymmetric part of the unit cell. The benzothiazine cycle adopted a distorted sofa conformation in molecules **A** and **B** (the puckering parameters [45] are: S = 0.65, Θ = 56.3, Ψ = 33.1 in molecule **A** and S = 0.65, Θ = 56.6, Ψ = 34.9 in molecule **B**). The C_(8)_ and S_(1)_ atoms deviated from the mean plane of the remaining atoms of this cycle by 0.36 Å and 0.95 Å in molecule **A** and 0.34 Å and 0.95 Å in molecule **B**. The nitrogen atom had a slightly pyramidal configuration in both molecules (the sum of the bond angles centered at the N_(1)_ atom is 352° in molecule **A** or 353° in molecule **B**). The C_(1)_–N_(1)_ bond was elongated (1.422(3) Å in **A** and 1.426(2) Å in **B**) as compared to the mean value [46] 1.371 Å.

The carboxylic fragment was turned in relation to the endocyclic double bond (the C_(7)_–C_(8)_–C_(9)_–O_(1)_ torsion angle was −52.9(3)° in **A** and 51.5(3)° in **B**) and the C_(7)_–C_(8)_ bond (1.344(3) Å in **A** and 1.335(2) Å in **B**) was elongated as compared to the mean value 1.326 Å) due to the repulsion between the vicinal methyl and ester substituents. The ethyl group of the ester substituent was localized in *sp*-conformation relatively to the C_(9)_–O_(1)_ bond and was almost orthogonal to the carboxylic fragment (the O_(1)_–C_(9)_–O_(2)_–C_(10)_ and C_(9)_–O_(2)_–C_(10)_–C_(11)_ torsion angles are −3.3(3)° and 82.0(2)° in molecule **A** and 0.5(3)° and −84.5(2)° in molecule **B**). The ethyl substituent at the N_(1)_ atom was turned relatively to the C_(1)_–N_(1)_ endocyclic bond (the C_(1)_–N_(1)_–C_(12)_–C_(13)_ torsion angle was 63.7(2)° in molecule **A** and −69.8(2)° in molecule **B**).

**Figure 3:**
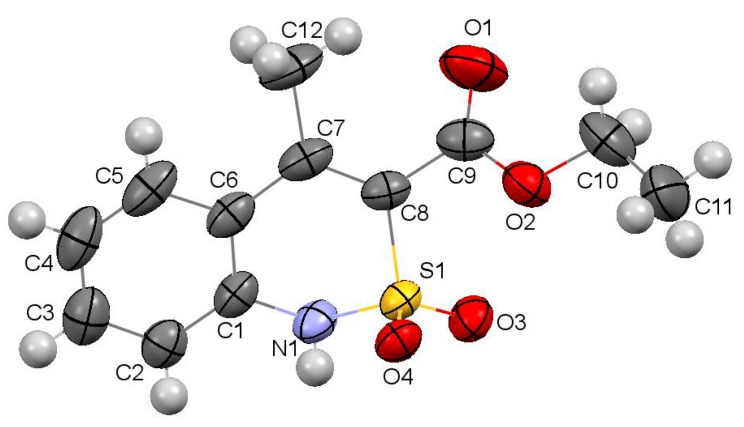
The spatial structure of ethyl ester **2** according to the X-ray diffraction data. The atoms are represented by thermal vibration ellipsoids of 50% probability.

According to the X-ray structural analysis, the second type of crystals from the mixture obtained after the alkylation of ester **1**—thick plates—were ethyl 4-methyl-2,2-dioxo-1*H*-2λ^6^,1-benzothiazine-3-carboxylate (**2**) (Figure 3).

Unlike its *N*-ethyl-substituted analog **3**, ester **2** is readily soluble in the aqueous solution of sodium carbonate due to salt formation by the cyclic sulfamide group. This property was used for simple and effective separation of the mixture of esters **2** and **3**. Ester **2**, obtained after the acidification of the alkaline solution, was again recrystallized in its pure form from the same solvent, i.e., ethanol. Surprisingly, this time the crystal habit changed—the crystals became thin slices. Moreover, X-ray diffraction analysis showed that the changes affected not only the appearance, but also the crystal structure. In other words, ester **2** was able to crystallize in two polymorphic modifications: in the presence of compound **3** it formed a monoclinic **2M** form with a spatial Рс group, and in its pure form it formed an orthorhombic **2O** form with a spatial Pbca group. Comparison of the geometrical characteristics of the molecule in different crystalline forms of ester **2** showed that their molecular (but not crystalline) structure was almost the same. In both cases, the benzothiazine cycle was in conformation of a distorted sofa, as evidenced by the parameters of puckering and deviation of S_(1)_and C_(8)_ atoms from the mean square plane of other atoms of the cycle (see Table 1). The steric repulsion between the methyl group and the ester substituent (shortened intramolecular contact C_(12)_…O_(1)_ compared to the sum of the van der Waals radii [47] 3.00 Å) lead to the elongation of the C_(7)_–C_(8)_ bond and the turn of the ethoxycarbonyl fragment with respect to the endocyclic double bond (C_(7)_–C_(8)_–C_(9)_–O_(1)_ torsion angle). The ethyl group is in an *ар*- conformation with respect to the C_(8)_–C_(9)_ bond and somewhat turned in relation to C_(9)_–O_(2)_ bond (C_(8)_–C_(9)_–O_(2)_–C_(10)_ and C_(9)_–O_(2)_–C_(10)_–C_(11)_ torsion angles, Table 1).

Interestingly, all our attempts to obtain a monoclinic polymorph modification **2M** by the homogeneous crystallization of pure ester **2** from different solvents (ethanol, ethyl acetate, aqueous *N,N*-dimethylformamide or methylene chloride) failed; furthermore, an exclusively orthorhombic form **2O** was always isolated. For some yet unknown reasons, the formation of the monoclinic form **2M** appeared to be possible only under conditions of heterogeneous crystallization, i.e., with the obligatory joint presence of foreign particles of *N*-ethyl-substituted ester **3** in the solution. It is quite difficult (if possible at all) to provide a theoretical explanation for this fact, which was accidentally discovered. Therefore, such findings often remain either a carefully guarded secret or a daily practice, but, unfortunately, they do not have any scientific background [48]. However, currently we were presented with the solution to the practical problem of the isolation of the monoclinic form **2M** in its pure form, which is of interest for pharmacological tests. It is clear that the method of separation of the mixture of esters **2М** and **3** described above is unsuitable for this, since it involves dissolving the target product. Thus, it was found that the impurity of *N*-ethyl-substituted ester **3** can be transferred into a solution, but the monoclinic form **2M** is left unchanged in a crystalline form using hot hexane (see Section 2).

**Figure 4:**
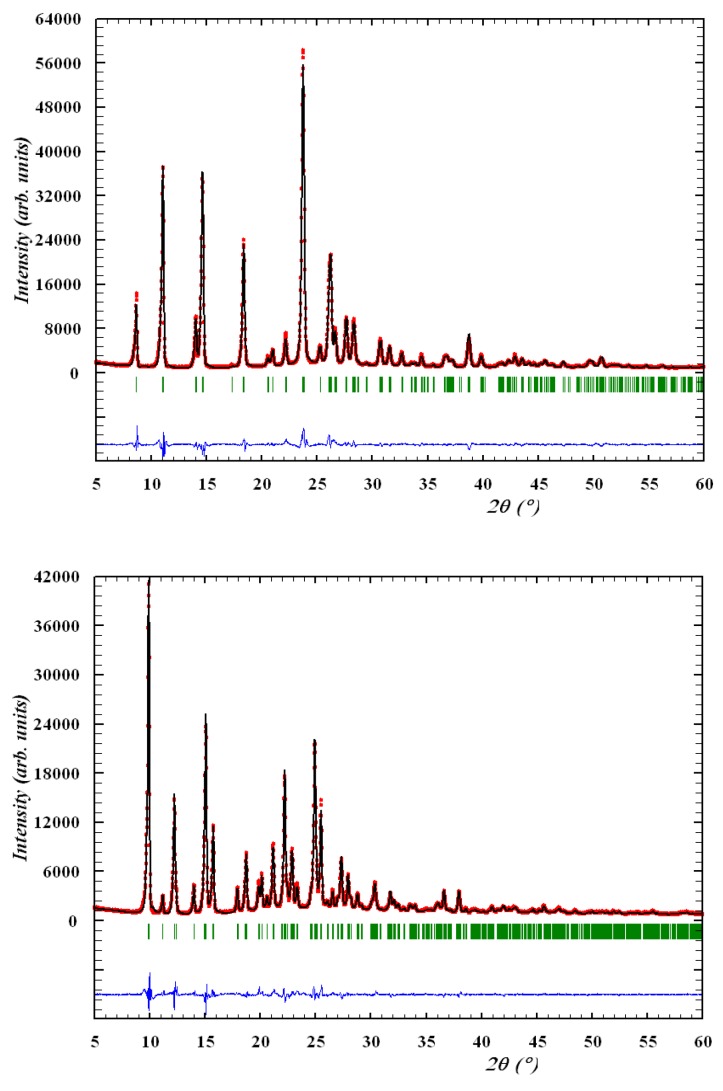
X-ray powder patterns of the monoclinic **2M** (**top**) and orthorhombic **2M** (**down**) forms of ester **2** and their Rietveld refinement: *red*—experimental X-ray diffraction patterns, *black*—calculated X-ray diffraction patterns, *green*—Bregg position of diffraction maximum, *blue*—difference between experimental and calculated intensity values in each point.

The phase purity of crystal forms is known to be very important for pharmaceutical study. Any impurity of by-product or other crystal form (polymorphic modification, hydrate, solvate, etc.) leads to unclear or wrong data regarding the biological activity. The phase purity of the monoclinic and orthorhombic modifications of ester **2** was confirmed by powder diffraction study (Figure 4). The data obtained from the powder diffraction experiments were compared to the data calculated from the single crystal structures and showed clearly that both polymorphic modifications were pure and did not contain any impurities. Thus, it may be expected that the biological experiment was informative and reasonably carried out.

### 3.2. Evaluation of the Anti-Inflammatory and Analgesic Activity

The analysis of the results of our tests (Table 2 and Table 3) suggests that the dialkylation product of salt **1**, i.e.,ethyl 1-ethyl-4-methyl-2,2-dioxo-1*H*-2λ^6^,1-benzothiazine-3-carboxylate (**3**), is of little interest as an object for further pharmacological study. The basis for this conclusion was a very moderate analgesic and an extremely weakly expressed anti-inflammatory effect demonstrated by this compound in the animal experiments. However, when revealing the regularities of the “structure–activity” relationship, which are important for further purposeful searches for new biologically active substances, such information is also valuable.

Much more attention should be paid to the study and comparison of the biological properties of the different crystal modifications of ester **2**. These studies have recently aroused keen interest among a wide range of researchers engaged in the creation of drugs, since they involve the intellectual field called polymorphism of crystals, which is insufficiently studied, still poorly understood and not always predictable. Indeed, the crystal modifications of ethyl 4-methyl-2,2-dioxo-1*H*-2λ^6^,1-benzothiazine-3-carboxylate (**2**) obtained were surprisingly different in their biological properties (Table 2 and Table 3), although, in fact, they are the same substance. Thus, if the orthorhombic form **2O,** with a sufficiently pronounced analgesic action, had a very weak anti-inflammatory effect, then the monoclinic form **2M** under the same conditions and in the same dose was both a powerful analgesic and anti-exudative agent significantly exceeding not only Piroxicam, but its more active analog Meloxicam by these indicators. All this gives grounds for a detailed study of the features of the crystal structure of esters **2M** and **2O**, as an obvious factor that largely contributes to the observed therapeutic effects. In comparison, in the case of *N-*benzyl-4-hydroxy-1-methyl-2,2-dioxo-1*H*-2λ^6^,1-benzothiazine-3-carboxamide and the solvates of its 4-*O*-sodium salt, this factor was the spatial structure [49].

### 3.3. The Crystal Structure Study

**Figure 5:**
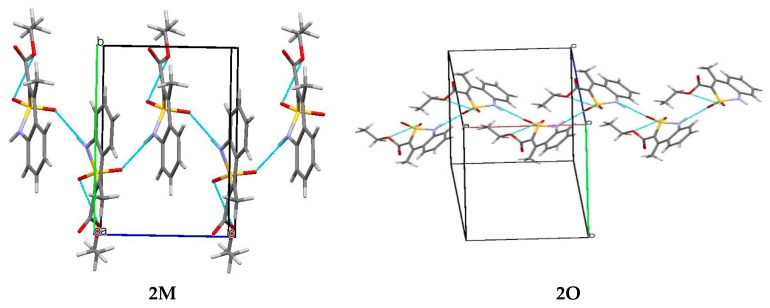
The chains of ethyl 4-methyl-2,2-dioxo-1*H*-2λ^6^,1-benzothiazine-3-carboxylate molecules in the monoclinic **2M** (**left**) and orthorhombic **2O** (**right**) polymorphic forms.

As mentioned above, the spatial structure of ester **2** was almost same in the two polymorphic forms **2M** and **2O**. In contrast, the crystal structure of the two polymorphic forms was very different. In the monoclinic crystals, the **2M** molecules of ethyl 4-methyl-2,2-dioxo-1*H*-2λ^6^,1-benzothiazine-3-carboxylate formed the infinite chains (Figure 5, left) along the crystallographic direction [001]. The molecules within the chain are bound by the N_(1)_–H…O_(4′)_ intermolecular hydrogen bonds (*x*, 1 −*y*, −0.5 + *z*; H…O 2.14 Å, N–H…O 165°) and stacking interactions (the distance between aromatic rings was 3.47 Å), simultaneously. There were not any specific interactions between the molecules belonging to the neighboring chains. In the case of the orthorhombic modification **2O** (Figure 5, right), the molecules of the ester formed the infinite chains in the crystallographic direction [100] and were bound only by the N_(1)_–H…O_(4′)_ intermolecular hydrogen bonds (−0.5 + *x*, 0.5 − *y*, 1 − *z*) (H…O 2.10 Å, N–H…O 161°) within the chain. Stacking interactions were observed between the molecules of the neighboring chains (the distance between the aromatic rings was 3.36 Å). The geometric characteristics of the hydrogen bonds and stacking interactions allowed us to presume that the molecules of ethyl 4-methyl-2,2-dioxo-1*H*-2λ^6^,1-benzothiazine-3-carboxylate were bound slightly stronger in the form **2O**.

It should be noted that the analysis of the geometric characteristics of intermolecular interactions does not allow discussion of the interaction energies between molecules in the crystal in full measure. At the same time, the difference in the biological activity of the two polymorphic modifications of one compound with the same spatial structure can be caused by the rate of the crystal structure destruction and therefore depends on the interactions between molecules.

The earlier suggested method of crystal structure analysis based of the comparison of the pairwise interaction energies between molecules in the crystal phase [37,50], allows one to turn from the qualitative analysis of the obtained intermolecular interactions to the quantitative estimation of the full interaction energies between molecules. According to this method, any molecule in a crystal may be chosen as the basic molecule M_0_, which is a building unit of a crystal. The first coordination sphere of this molecule is determined using a standard option and contains a quantity of neighboring molecules М_1,2…n_, interacting with the basic M_0_. The interaction energies within the dimers М_0_–М_1_, М_0_–М_2,_ М_0_–М_3_ … М_0_–М_n_ are estimated using quantum-chemical calculations. The normalization of the obtained values of interaction energies relatively the strongest for this basic molecule interaction energy causes the replacement of the absolute energies values by their relative values which are used for the construction of the energy-vector diagram (EVD). This diagram is an image of the basic molecules reflecting its interaction energies with all neighboring molecules.

**Figure 6:**
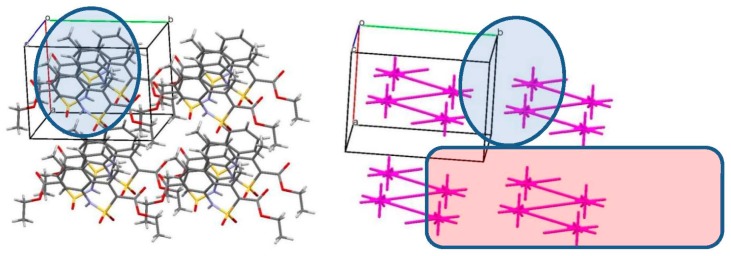
The packing of ester **2** in the monoclinic polymorphic form **2M** presented as the packing of molecules (**left**) and packing of energy-vector diagrams (**right**). The primary basic structural motif (chain) is highlighted in blue. The secondary basic structural motif (layer) is highlighted in pink.

The application of the suggested method to the monoclinic polymorphic modification **2M** revealed that the first coordination sphere of the basic molecule contained 14 neighboring molecules bound to it by some energy and symmetry operations. The summary interaction energy of the basic molecule with all those belonging to its first coordination sphere was −78.1 kcal/mol. The strongest interactions were observed with two neighboring molecules causing the formation of two identical dimers **2M_1** and **2M_2** with equal interaction energies (Table 4). This caused the formation of a zigzag chain (Figure 6) in the crystallographic direction [001]. The interaction energy of the basic molecule with the two neighboring molecules within this chain was −35.9 kcal/mol or 46.0% of the full interaction energy with all molecules belonging to its first coordination sphere. The molecules within the chain were bound by the N–H…O intermolecular hydrogen bonds and the stacking interaction, as it was detected using the traditional analysis of the intermolecular interactions in the crystal phase. The separated chain can be considered the primary basic structural motif of the monoclinic polymorphic modification of ethyl 4-methyl-2,2-dioxo-1*H*-2λ^6^,1-benzothiazine-3-carboxylate.

It should be noted that the traditional analysis of the crystal packing for this structure is confined by the separation out of the chain. It is caused by the absence of specific interactions between molecules belonging to the neighboring chains. The application of the quantum-chemical calculations allowed us to estimate the interaction energies between the molecules bound by non-specific interactions. As with the **2M** crystal, it was shown that the interaction energies between molecules of the neighboring chains were not equal. The chains arranged within the plane parallel to the crystallographic plane (011) interacted more strongly (the interaction energy between the molecules belonging neighboring chains was −17.6 kcal/mol or 22.5% from the total interaction energy within the first coordination sphere). As a result, the layer was formed as the second basic structural motif. The interaction energy of the basic molecule with all the neighboring molecules within the layer was −53.5 kcal/mol (68.5%), while the interaction energy with molecules of the neighboring layer was only −9.9 kcal/mol (12.7%). Thus, the monoclinic crystal structure of ester **2** was built by the molecules as building units and may be divided on two levels of organization from the viewpoint of interaction energies between molecules: chains as the primary level of organization and layers of strongly interacting chains as the secondary level of organization.

**Figure 7:**
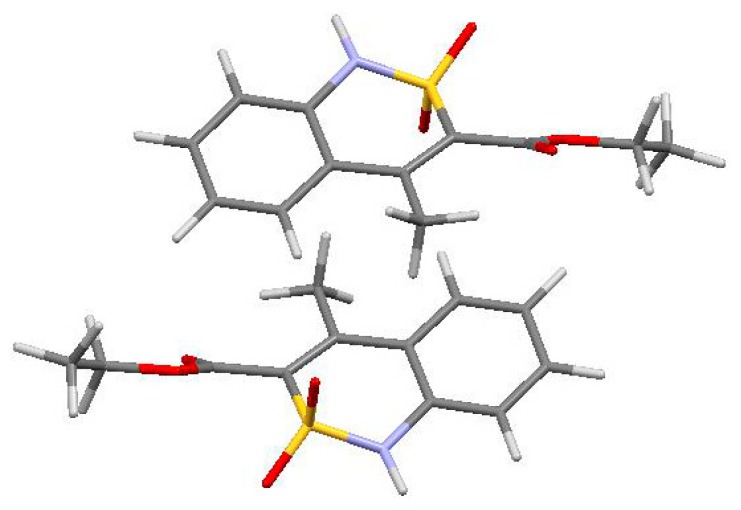
Dimer of the molecules of ester **2**, which is the building unit of the orthorhombic polymorphic form **2O**.

The analysis of the orthorhombic polymorphic modification **2O** showed that the first coordination sphere of the ester **2** contained 14 neighboring molecules, similar the monoclinic modification. However, the total interaction energy of the basic molecule with all those in its first coordination sphere was slightly weaker compared to the monoclinic modification (−73.9 kcal/mol). In contrast to the monoclinic crystal form, the basic molecules formed the strongest interaction with only one neighboring molecule in the orthorhombic crystal (Table 4). Taking into account that the interaction energy in the dimer **2O_1** is significantly stronger compared to the next dimer, we may assert that the building unit in crystal **2O** was the dimer (Figure 7) rather than the molecule. The molecules in such a complex building unit are bound by very weak C–H…O and C–H… π hydrogen bonds and mainly by a lot of different types of non-specific interactions (total electrostatic, dispersion, polarization, etc.). It should be noted that this strongly bound dimer was not separated out by the traditional analysis of the intermolecular interactions in the crystal.

**Figure 8:**
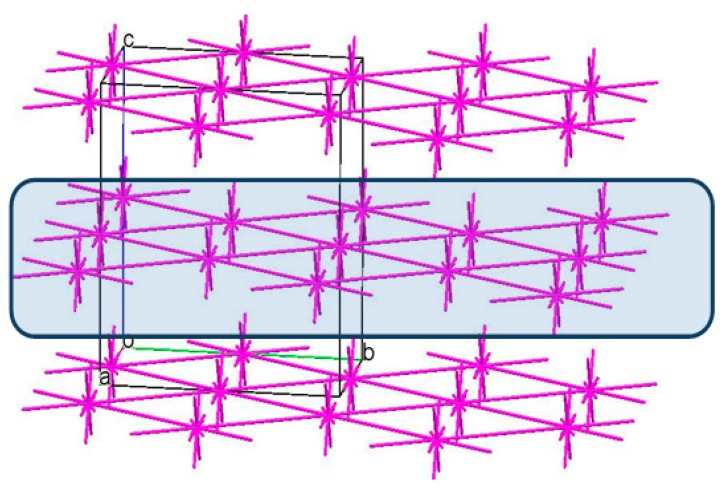
The packing of the dimers **2O_1** in the orthorhombic polymorphic form presented as the packing of energy-vector diagrams. The layer as the primary basis structural motif is highlighted in blue.

In the next stage of the orthorhombic crystal structure analysis, the first coordination sphere of the dimer **2O_1** was constructed. The interaction energies with all 14 neighboring dimers were calculated. The analysis of the interaction energies showed that the basic dimer formed the four strongest interactions with the neighboring dimers. It caused the formation of the layer parallel to the crystallographic plane (110) as the primary basic structural motif (Figure 8). The neighboring dimers were bound by the N–H…O intermolecular hydrogen bonds within the layer and the interaction energy of the basic dimer within the layer was −85.4 kcal/mol (70.2% of the total interaction energy). The interaction energy with the neighboring layers was much weaker: −15.0 kcal/mol or 12.4%. Thus, the orthorhombic crystal structure of the ester **2** was built by the dimers of strongly bound molecules as building units and only one level of organization was able to be separated out.

Thus, the analysis of interaction energies between the molecules showed that two polymorphic modifications of the ester **2** had different building units (the molecule in monoclinic **2M** and the dimer of the molecules in orthorhombic **2O**), but a similar layered type of crystal packing. Moreover, the relations between the interaction energies within the layer and between the neighboring layers were very close in two polymorphic modifications. This allows us to presume that the difference between the two modifications might be minimal in the first stage of crystal destruction. However, the layer was homogeneous and was made of strongly bound dimers in the orthorhombic form, while the layer of the monoclinic form was not homogeneous and was made of chains. We may presume that this difference was the factor causing the best bioavailability, dissolution rate and/or solubility of the monoclinic form **2M**, due to the easier destruction of the layers.

## 4. Conclusions

The reaction of sodium4-methyl-2,2-dioxo-1*H*-2λ^6^,1-benzothiazine-3-carboxylate monohydrate and an equivalent amount of iodoethane in DMSO solution was studied. It was found that not only the oxygen atom of the carboxyl group is subjected to alkylation, but to a significant extent a heteroatom of nitrogen. As a result, two esters—ethyl 4-methyl-2,2-dioxo-1*H*-2λ^6^,1-benzothiazine-3-carboxylate and its 1-ethyl-substituted analog—were isolated as products of the reaction studied. In all cases, the true direction of the alkylation was determined by X-ray diffraction analysis. Moreover, the ability to exist in two polymorphic forms—monoclinic and orthorhombic—was found in ethyl 4-methyl-2,2-dioxo-1*H*-2λ^6^,1-benzothiazine-3-carboxylate. According to the data from the pharmacological tests, the monoclinic form showed strong analgesic and anti-inflammatory effects, whereas the orthorhombic form appeared to be relatively less active. Based on a detailed analysis of the molecular and crystal structure of two polymorphic forms of ethyl 4-methyl-2,2-dioxo-1*H*-2λ^6^,1-benzothiazine-3-carboxylate, the possible causes of the much higher activity of the monoclinic crystal form were suggested.

## Figures and Tables

**Table 1:** Some geometric characteristics of the polymorphic form ester **2**.

Entry	Parameter	Monoclinic (2M)	Orthorhombic (2O)
1	S	0.58	0.63
2	Θ, deg	49.8	54.2
3	Ψ, deg	37.5	31.4
4	S_(1)_, Å	0.78	0.89
5	C_(8)_, Å	0.22	0.33
6	C_(7)_–C_(8)_, Å	1.361(4)	1.362(4)
7	N_(1)_–C_(1)_, Å	1.412(4)	1.398(3)
8	C_(7)_–C_(8)_–C_(9)_–O_(1)_, deg	−36.1(5)	−37.8(5)
9	C_(8)_–C_(9)_–O_(2)_–C_(10)_, deg	177.9(3)	−179.8(2)
10	C_(9)_–O_(2)_–C_(10)_–C_(11)_, deg	−160.4(4)	173.3(3)
11	C_(12)_…O_(1)_, Å	2.94	2.96

**Table 2:**
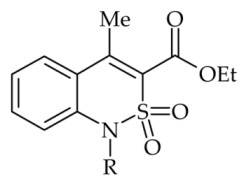
The anti-inflammatory activity of esters **2M**, **2O**, **3**, and reference drugs.

Entry	Product	R	Volume of Damaged Extremity (mm^3^)	Volume of Non-Damaged Extremity (mm^3^)	Δ Volume (Volume Increase)	Anti-Inflammatory Activity, Compared to Control (%)
1	**2M**	H	377.1 ± 29.9	318.3 ± 20.4	58.8 ± 5.8 ^1,2,3^	85.8
2	**2O**	H	736.2 ± 23.3	449.5 ± 23.9	286.7 ± 8.6 ^1,2,3^	30.7
3	3	Et	743.5 ± 32.0	455.2 ± 21.2	288.2 ± 8.0 ^1,2,3^	30.3
4	Piroxicam	−	566.7 ± 160.4	342.8 ± 19.8	223.8 ± 6.1 ^1^	45.9
5	Meloxicam	−	492.6 ± 39.3	361.2 ± 26.1	131.3 ± 8.0 ^1^	68.3
6	Control	−	768.7 ± 27.3	354.9 ± 11.6	413.7 ± 32.2	0

^1^ Differences statistically significant for *p* ≤ 0.05 vs. control. ^2^ Differences statistically significant for *p* ≤ 0.05 vs. Piroxicam. ^3^ Differences statistically significant for *p* ≤ 0.05 vs. Meloxicam.

**Table 3:** The Analgesic Activity of Esters **2M**, **2O**, **3**, and Reference Drugs.

Entry	Product	R	Pain Threshold on Damaged Extremity (g/mm^2^)	Pain Threshold on Non-Damaged Extremity (g/mm^2^)	Δ Pain Threshold	Analgesic Activity, Compared to Control (%)
1	**2M**	H	370.0 ± 15.2	340.0 ± 17.0	30.0 ± 4.5 ^1,2,3^	90.6
2	**2O**	H	344.0 ± 28.8	236.0 ± 11.4	108.0 ± 8.6 ^1,2,3^	66.0
3	**3**	Et	380.0 ± 10.5	220.0 ± 13.4	160.0 ± 10.5 ^1,2,3^	49.7
4	Piroxicam	−	504.0 ± 18.1	340.0 ± 15.2	164.0 ± 8.1 ^1^	48.4
5	Meloxicam	−	414.0 ± 19.6	326.0 ± 26.4	88.0 ± 11.6 ^1^	72.3
6	Control	−	593.0 ± 56.3	275.0 ± 32.1	318.0 ± 34.9	0

^1^ Differences statistically significant for *p* ≤ 0.05 vs. control. ^2^ Differences statistically significant for *p* ≤ 0.05 vs. Piroxicam. ^3^ Differences statistically significant for *p* ≤ 0.05 vs. Meloxicam.

**Table 4:** Symmetry codes, bonding type, interaction energy of the basic molecule with neighboring ones (E_int_, kcal mol^−1^), with the highest values (more than 5% of the total interaction energy) and the contribution of this energy to the total interaction energy (%) in the crystals **2M** and **2O**.

Dimer	Symmetry Operation	E_int_ (kcal · mol^−1^)	Contribution to the Total Interaction Energy (%)	Interaction Type
Crystals of **2M**				
**2M_1**	*x*, 1 − *y*, 0.5 + *z*	−17.95	22.98	N–H…O, stacking
**2M_2**	*x*, 1 − *y*, −0.5 + *z*	−17.95	22.98	N–H…O, stacking
**2M_3**	*x*, 2 − *y*, 0.5 + *z*	−8.78	11.24	non-specific
**2M_4**	*x*, 2 − *y*, −0.5 + *z*	−8.78	11.24	non-specific
**2M_5**	1 + *x*, *y*, *z*	−5.86	7.50	non-specific
**2M_6**	−1 + *x*, *y*, *z*	−5.86	7.50	non-specific
Crystals of **2O**, building unit is a molecule				
**2O_1**	1 − *x*, 1 − *y*, 1 − *z*	−16.56	22.41	C–H…O, C–H…π
**2O_2**	0.5 + *x*, 0.5 − *y*, 1 − *z*	−11.51	15.58	N–H…O
**2O_3**	−0.5 + *x*, 0.5 − *y*, 1 − *z*	−11.51	15.58	N–H…O
**2O_4**	−*x*, 1 − *y*, 1 − *z*	−7.98	10.81	stacking
**2O_5**	0.5 + *x*, *y*, 0.5 − *z*	−5.03	6.81	C–H…π
**2O_6**	−0.5 + *x*, *y*, 0.5 − *z*	−5.03	6.81	C–H…π
Crystals of **2O**, building unit is a dimer				
**2O_d_1**	1/2 + *x*, 1/2 − *y*, 1 − *z*	−16.11	13.25	N–H…O
**2O_d_2**	1/2 + *x*, 3/2 − *y*, 1 − *z*	−16.11	13.25	N–H…O
**2O_d_3**	−1/2 + *x*, 1/2 − *y*, 1 − *z*	−16.11	13.25	N–H…O
**2O_d_4**	−1/2 + *x*, 3/2 − *y*, 1 − *z*	−16.11	13.25	N–H…O
**2O_d_5**	1 + *x*, *y*, *z*	−10.47	8.61	stacking
**2O_d_6**	−1 + *x*, *y*, *z*	−10.47	8.61	stacking
**2O_d_7**	0.5 − *x*, 1 − *y*, 0.5 + *z*	−7.52	6.18	C–H…O, C–H…π
**2O_d_8**	1/2 − *x*, 1 − *y*, −1/2 + *z*	−7.52	6.18	C–H…O, C–H…π
**2O_d_9**	3/2 − *x*, 1 − *y*, 1/2 + *z*	−7.52	6.18	C–H…O, C–H…π
**2O_d_10**	3/2 − *x*, 1 − *y*, −1/2 + *z*	−7.52	6.18	C–H…O, C–H…π

## References

[1] Kleemann A., Engel J., Kutscher B., Reichert D. (2008). Pharmaceutical Substances: Syntheses, Patents, Applications of the Most Relevant APIs.

[2] O’Neil M.J., Heckelman P.E., Koch C.B., Roman K.J. (2006). The Merck Index: An Encyclopedia of Chemicals, Drugs, and Biologicals.

[3] Kuznetsov S.G., Chigareva S.M., Ramsh S.M. (1981). Prodrugs: Chemical aspect. Results Sci. Technol. VINITI Ser. Org. Chem..

[4] Fukami T., Yokoi T. (2012). The emerging role of human esterases. Drug Metab. Pharmacokinet..

[5] Sakakura A., Kawajiri K., Ohkubo T., Kosugi Y., Ishihara K. (2007). Widely useful DMAP-catalyzed esterification under auxiliary base- and solvent-free conditions. J. Am. Chem. Soc..

[6] Pittelkow M., Kamounah F.S., Boas U., Pedersen B., Christensen J.B. (2004). TFFH as an excellent reagent for acylation of alcohols, thiols and dithiocarbamates. Synthesis.

[7] Dhimitruka I., Santa Lucia J. (2006). Investigation of the Yamaguchi esterification mechanism. Synthesis of a Lux-S enzyme inhibitor using an improved esterification method. Org. Lett..

[8] Bartoli G., Bosco M., Carlone A., Dalpozzo R., Marcantoni E., Melchiorre P., Sambri L. (2007). Reaction of dicarbonates with carboxylic acids catalyzed by weak Lewis acids: General method for the synthesis of anhydrides and esters. Synthesis.

[9] Dev D., Palakurthy N.B., Thalluri K., Chandra J., Mandal B. (2014). Ethyl 2-cyano-2-(2-nitrobenzene-sulfonyloxyimino)acetate (*o*-NosylOXY): A recyclable coupling reagent for racemization-free synthesis of peptide, amide, hydroxamate, and ester. J. Org. Chem..

[10] Chen Z., Wen Y., Fu Y., Chen H., Ye M., Luo G. (2017). Graphene oxide: An efficient acid catalyst for the construction of esters from acids and alcohols. Synlett.

[11] Minakawa M., Baek H., Yamada Y.M.A., Han J.W., Uozumi Y. (2013). Direct dehydrative esterification of alcohols and carboxylic acids with a macroporous polymeric acid catalyst. Org. Lett..

[12] Kim Y.-H., Han J., Jung B.Y., Baek H., Yamada Y.M.A., Uozumi Y., Lee Y.-S. (2016). Production of valuable esters from oleic acid with a porous polymeric acid catalyst without water removal. Synlett.

[13] Jaita S., Phakhodee W., Pattarawarapan M. (2015). Ultrasound-assisted methyl esterification of carboxylic acids catalyzed by polymer-supported triphenylphosphine. Synlett.

[14] Huang Z., Reilly J.R., Buckle R.N. (2007). An efficient synthesis of amides and esters via triacyloxyboranes. Synlett.

[15] Chakraborti A.K., Singh B., Chankeshwara S.V., Patel A.R. (2009). Protic acid immobilized on solid support as an extremely efficient recyclable catalyst system for a direct and atom economical esterification of carboxylic acids with alcohols. J. Org. Chem..

[16] Srinivas K.V.N.S., Mahender I., Das B. (2003). Silica Chloride: A versatile heterogeneous catalyst for esterification and transesterification. Synthesis.

[17] Wang Y., Aleiwi B.A., Wang Q., Kurosu M. (2012). Selective esterifications of primary alcohols in a water-containing solvent. Org. Lett..

[18] Twibanire J.K., Grindley T.B. (2011). Efficient and controllably selective preparation of esters using uronium-based coupling agents. Org. Lett..

[19] Heller S.T., Sarpong R. (2010). Chemoselective esterification and amidation of carboxylic acids with imidazole carbamates and ureas. Org. Lett..

[20] Velusamy S., Borpuzari S., Punniyamurthy T. (2005). Cobalt(II)-catalyzed direct acetylation of alcohols with acetic acid. Tetrahedron.

[21] Sharghi H., Hosseini Sarvari M. (2003). Al_2_O_3_/MeSO_3_H (AMA) as a new reagent with high selective ability for monoesterification of diols. Tetrahedron.

[22] Hong X., Harmata M., Gribble G.W., Joule J.A. (2008). Recent Progress in the Chemistry of 2,1-Benzothiazines. Progress in Heterocyclic Chemistry.

[23] Azotla-Cruz L., Lijanova I.V., Ukrainets I.V., Likhanova N.V., Olivares-Xometl O., Bereznyakova N.L. (2017). New synthesis, structure and analgesic properties of methyl 1-R-4-methyl-2,2-dioxo-1*H*-2λ^6^,1- benzothiazine-3-carboxylates. Sci. Pharm..

[24] Ukrainets I.V., Petrushova L.A., Dzyubenko S.P. (2013). 2,1-Benzothiazine 2,2-dioxides. 1. Synthesis, structure, and analgesic activity of 1-R-4-hydroxy-2,2-dioxo-1*H*-2λ^6^,1-benzothiazine-3-carboxylic acid esters. Chem. Heterocycl. Compd..

[25] Ukrainets I.V., Petrushova L.A., Sim G., Grinevich L.A.S.P. (2017). Synthesis and molecular structure of ethyl-4-hydroxy-1-phenyl-2,2-dioxo-1*H*-2λ^6^,1-benzothiazine-3-carboxylate. Pharm. Chem. J..

[26] Ukrainets I.V., Hamza G.M., Burian A.A., Shishkina S.V., Voloshchuk N.I., Malchenko O.V. (2018). 4-Methyl-2,2-dioxo-1*H*-2λ^6^,1-benzothiazine-3-carboxylic acid. Peculiarities of preparation, structure, and biological properties. Sci. Pharm..

[27] Sheldrick G.M. (2008). A short history of SHELX. Acta Crystallogr. Sect. A Found. Crystallogr..

[28] Cambridge Crystallographic Data Center Request Quoting via: CCDC1835802. www.ccdc.cam.ac.uk/conts/retrieving.html.

[29] Cambridge Crystallographic Data Center Request Quoting via: CCDC1835803. www.ccdc.cam.ac.uk/conts/retrieving.html.

[30] Cambridge Crystallographic Data Center Request Quoting via: CCDC1835804. www.ccdc.cam.ac.uk/conts/retrieving.html.

[31] Rodriguez-Carvajal J. (2010). FullProf.

[32] Rodriguez-Carvajal J., Roisnel T. (2004). WinPLOTR.

[33] Rodriguez-Carvajal J., Roisnel T. (1998). FullProf.98 and WinPLOTR New Windows 99/NT Applications for Diffraction, Commission for Powder Diffraction.

[34] Ukrainian Law No. 3447-IV. On Protection of Animals from Severe Treatment. http://zakon2.rada.gov.ua/laws/show/3447-15.

[35] Vogel H.G. (2008). Drug Discovery and Evaluation: Pharmacological Assays.

[36] Gregory N.S., Harris A.L., Robinson C.R., Dougherty P.M., Fuchs P.N., Sluka K.A. (2013). An overview of animal models of pain: Disease models and outcome measures. J. Pain..

[37] Shishkin O.V., Dyakonenko V.V., Maleev A.V. (2012). Supramolecular architecture of crystals of fused hydrocarbons based on topology of intermolecular interactions. CrystEngComm.

[38] Macrae C.F., Bruno I.J., Chisholm J.A., Edgington P.R., McCabe P., Pidcock E., Rodriquez-Monge L., Taylor R., van de Streek J., Wood P.A. (2008). *Mercury CSD 2.0*—New features for the visualization and investigation of crystal structures. J. Appl. Cryst..

[39] Coppens P. (1972). The use of a polarized hydrogen atom in X-ray structure refinement. Acta Cryst. B.

[40] Grimme S. (2006). Semiempirical GGA-type density functional constructed with a long-range dispersion correction. J. Comput. Chem..

[41] Grimme S., Ehrlich S., Goerigk L. (2011). Effect of the damping function in dispersion corrected density functional theory. J. Comput. Chem..

[42] Grimme S., Antony J., Ehrlich S., Krieg H. (2010). A consistent and accurate *ab initio* parametrization of density functional dispersion correction (DFT-D) for the 94 elements H-Pu. J. Chem. Phys..

[43] Boys S., Bernardi F. (1970). The calculation of small molecular interactions by the differences of separate total energies. Some procedures with reduced errors. Mol. Phys..

[44] Neese F. (2012). The ORCA program system. Wiley Interdiscip. Rev. Comput. Mol. Sci..

[45] Zefirov N.S., Palyulin V.A., Dashevskaya E.E. (1990). Stereochemical studies. XXXIV. Quantitative description of ring puckering via torsional angles. The case of six-membered rings. J. Phys. Org. Chem..

[46] Orpen A.G., Brammer L., Allen F.H., Kennard O., Watson D.G., Taylor R., Burgi H.-B., Dunitz J.D. (1994). Typical interatomic distances in organic compounds and organometallic compounds and coordination complexes of the d- and f-block metals. Structure Correlation.

[47] Zefirov Y.V. (1997). Reduced intermolecular contacts and specific interactions in molecular crystals. Crystallogr. Rep..

[48] Bernstein J. (2002). Polymorphism in Molecular Crystals.

[49] Ukrainets I.V., Petrushova L.A., Shishkina S.V., Grinevich L.A., Sim G. (2016). Synthesis, spatial structure and analgesic activity of sodium 3-benzylaminocarbonyl-1-methyl-2,2-dioxo-1H-2λ^6^,1-benzothiazin-4-olate solvates. Sci. Pharm..

[50] Shishkin O.V., Zubatyuk R.I., Shishkina S.V., Dyakonenko V.V., Medviediev V.V. (2014). Role of supramolecular synthons in the formation of the supramolecular architecture of molecular crystals revisited from an energetic viewpoint. PhysChemChemPhys.

